# From Community Benefits to Vulnerabilities: Reverse-Logic Analysis of Nature-Based Solution Treescapes Across Europe

**DOI:** 10.3390/ijerph23060691

**Published:** 2026-05-23

**Authors:** Timothy Pittaway, Leanne Townsend, Claire Hardy

**Affiliations:** Social, Economic and Geographical Sciences (SEGS), The James Hutton Institute, Craigiebuckler, Aberdeen AB15 8QH, UK

**Keywords:** nature-based solutions, NBS treescapes, climate change, socio-ecological vulnerability, environmental regulation, public health, biodiversity support

## Abstract

**Highlights:**

**Public health relevance—How does this work relate to a public health issue?**

- Urban treescapes mitigate socio-ecological vulnerabilities, particularly extreme heat and limited access to green space. These interventions address systemic inequities that disproportionately impact population health.
- This study uses a novel reverse-logic methodology to map treescape benefits to underlying health deficits. It provides a framework for identifying region-specific vulnerabilities.

**Public health significance—Why is this work of significance to public health?**

- This study introduces a novel reverse-logic methodology that links observed benefits of the treescape directly to underlying socio-ecological and health vulnerabilities.
- It demonstrates that environmental stressors disproportionately affect human well-being when social inequities limit equitable access to high-quality green infrastructure.

**Public health implications—What are the key implications or messages for practitioners, policy makers and/or researchers in public health?**

- Public health and urban planning policies should adopt place-sensitive, equity-driven treescape designs tailored to specific regional climatic and health vulnerabilities.
- Interventions should move beyond isolated hazard mitigation by integrating ecological and social strategies to address physical security, well-being, and social cohesion simultaneously.

**Abstract:**

Nature-based solutions (NBSs) involving tree-based interventions deliver multiple community benefits, yet evidence linking these benefits to underlying socio-ecological vulnerabilities remains limited. This study synthesised metadata from 131 European treescape NBS case studies spanning eight biogeographical regions using reverse-logic, thematic qualitative analysis. Case studies were identified via adapted PRISMA guidelines from open-access repositories, with community benefit themes categorised and mapped spatially across bioregions. The analysis revealed eleven principal community benefit categories and distinct region-specific patterns: Mediterranean interventions primarily mitigated extreme heat and drought vulnerabilities, whilst Alpine projects addressed slope stability and hazard reduction. The Continental and Atlantic regions emphasised social cohesion, recreational access, and the preservation of cultural heritage. The reverse-logic methodology successfully identified underlying socio-ecological vulnerabilities through systematic analysis of observed benefit profiles across diverse European contexts. This approach provides evidence-based guidance for designing location-sensitive treescape NBS that advance environmental research and public health objectives. The findings establish a methodological foundation for future assessments of NBS effectiveness and for refining location-specific treescape interventions that address community vulnerabilities and enhance adaptive capacity.

## 1. Introduction

Nature-based solutions (NBSs), specifically tree-based green infrastructure, hereafter referred to as ‘treescapes’, offer critical interventions for public health and community well-being. This paper explores how these interventions address socio-ecological vulnerabilities. The underlying effectiveness of these spaces can be understood through the biophilia hypothesis, which posits that humans possess an inherent, evolutionarily derived affinity for nature that shapes our psychological and behavioural responses to the environment [1]. This connection to nature is not merely aesthetic or recreational; it can have benefits in increased happiness [2]. Cervinka et al. [3] highlight the well-being benefits of woodland exposure, noting consistent improvements in emotional states, stress reduction, and mental restoration. Physiological effects included lowered cortisol levels, blood pressure, and heart rate, even after brief visits and regardless of physical activity. The review also emphasises the role of woodlands in promoting social inclusion, particularly for vulnerable groups, through community-based and therapeutic initiatives.

In the context of this study, NBS are defined as actions that protect, sustainably manage, and restore ecosystems to address societal challenges effectively. However, the practical implementation of these solutions faces significant global hurdles. Recent research, notably from the NL2120 programme, one of the world’s most extensive public–private collaborations on nature-based solutions, identifies the ‘lack of data on NBS benefits’ as the third most significant obstacle to scaling these interventions [4]. This data deficit, alongside challenges in monitoring and evaluation, underscores the urgent need for new analytical frameworks that can systematically evidence the value of green infrastructure.

Indicators commonly employed to evaluate the worth of trees focus mainly on tangible commodities, such as timber and non-timber forest products, sometimes overlooking non-material services, including recreation activities and public engagement, that contribute significantly to human well-being [5]. Advancements in indicator systems, such as the Montreal Criteria and Indicators, have emphasised the significance of considering a broader spectrum of forest values beyond solely tangible commodities, integrating all forest outputs important to society [6]. The limited scope of current assessment criteria underscores the need for broader metrics that encompass the diverse advantages of forests, including their recreational, cultural, and social significance. These newer approaches emphasise the need to incorporate non-market values, including ecosystem services such as conservation, into forest assessments to provide a more comprehensive understanding of forests’ worth and impact on sustainable human well-being. Physical activities in areas of tree cover significantly contribute to fitness and health by reducing the risk of chronic diseases and improving cardiovascular health, and can add value to and benefit the lives of people with various conditions, including dementia [7,8,9].

These activities also enhance mental well-being by lowering stress levels and improving mood. Time spent in nature has been shown to positively impact multiple health indicators, including mental health [10,11]. Consequently, these health benefits result in reduced public health costs and improved quality of life [12]. Recreation values of forested areas vary significantly depending on factors such as available facilities, types of activities, visit frequency, and proximity to populations. This variability underscores the need for tailored management practices that optimise the recreational potential and economic value of different treescapes [13]. Wooded areas offer spaces for social interaction, relaxation, and connection with nature, contributing to mental health and social well-being. These areas also foster social cohesion by creating inclusive environments that facilitate diverse community interactions [14].

Additionally, woodlands provide subsistence resources and opportunities for fruitful endeavours through activities such as foraging, with such practices often serving as a source of cultural heritage for local communities [15]. Urban green spaces, particularly during the COVID-19 pandemic, provided residents with mental and physical health benefits through visits that supported stress reduction and general well-being. The proximity and easy access of urban green spaces make them important neighbourhood resources [16]. Wooded areas serve as outdoor classrooms through initiatives such as Outdoor Woodland Learning in the United Kingdom, providing educational resources on nature, ecology, and conservation. The utilisation of woodlands by children and adolescents has the potential to be a significant factor in their growth and development, providing avenues for unrestricted recreational activities and experiencing nature [17].

The substantial body of research demonstrating benefits to human well-being, environmental health, and social cohesion in these green spaces reveals both remarkable consistency in findings and the complex interconnected nature of these systems. While evidence consistently shows positive outcomes across physical, mental, and social dimensions, the diversity of treescape types, management approaches, and socio-demographic contexts creates varied pathways through which these benefits are realised. It is therefore important to consider the range of benefits offered by community tree-based green infrastructure, those accessible arboreal environments located within or near communities, to understand their potential in supporting comprehensive human well-being and informing evidence-based management decisions that can optimise multiple benefits simultaneously.

This research represents an exploratory methodological attempt to systematically link the benefits of treescape NBS to vulnerabilities within socio-ecological environments. Unlike traditional approaches that often assume direct causal relationships between environmental interventions and problem-solving outcomes, this methodology adopts a framework that interrogates the complex, non-linear connections between what treescapes deliver in terms of benefits and what social–ecological vulnerabilities they unintentionally or purposely address.

While the existing literature and systematic reviews extensively catalogue the positive health and well-being outcomes of forest exposure, they often lack frameworks for identifying the specific socio-ecological deficits driving the need for such interventions. This study addresses this gap by formalising a reverse-logic methodology, shifting the analytical focus from merely quantifying benefits to actively identifying the underlying vulnerabilities those benefits mitigate.

## 2. Aim, Objectives and Research Questions

This study aims to advance understanding to (a) identify and categorise the primary community benefit types documented in European treescape NBS databases and repositories; (b) to spatially analyse and map the distribution patterns of community benefits associated with NBS treescape projects across Europe’s biogeographical regions; (c) to examine and characterise the underlying community and ecosystem vulnerabilities that treescape NBS benefits specifically address; and (d) to investigate and determine the factors that drive vulnerability differences across different biogeographical regions in relation to treescape NBS. These objectives follow a progressive logic from identification and categorisation through spatial analysis to investigation of socio-ecological vulnerability relationships.

To achieve these objectives, the following research questions guided the research process:

RQ1: What categories of community benefits are most frequently identified in European treescape NBS databases and repositories?

RQ2: How are the community benefits associated with NBS treescape projects distributed across Europe’s biogeographical regions?

RQ3: What are the underlying community and ecosystem vulnerabilities that the benefits associated with treescape NBS address?

RQ4: What are the determinants of vulnerability scenarios (clusters)?

## 3. Concepts and Definitions

### 3.1. Nature-Based Solutions

NBSs are “actions to protect, conserve, restore, sustainably use and manage natural or modified terrestrial, freshwater, coastal and marine ecosystems which address community, economic and environmental challenges effectively and adaptively, while simultaneously providing human well-being, ecosystem services, resilience and biodiversity benefits” [18]. In practice, NBSs draw inspiration from, are supported by, or mimic natural processes. Green infrastructure interventions, such as the restoration and sustainable management of forests, wetlands, and other ecosystems, harness these processes to address interconnected environmental, social, and economic challenges. Such approaches generate multiple co-benefits, including safeguarding ecological functions, enhancing biodiversity, improving water quality, strengthening climate resilience, and providing cost-effective solutions which bring positive impacts to local economies and communities [19]. NBSs are increasingly recognised as effective strategies for addressing various community challenges, including environmental, social, and economic issues. These solutions leverage natural processes and ecosystems to provide sustainable and resilient alternatives to conventional infrastructure and management practices. An NBS strengthens resilience at multiple levels by not separating human and natural systems into separate categories, and instead enhancing the interconnectedness and complexity necessary to support living systems and quality of life [20].

Despite the many potential benefits, NBSs have been met with criticism for various reasons. Remme and Haardstad [21] highlight the tendency for NBS language to romanticise both the nature and participatory aspects of NBSs, arguing that in practice, levels of community participation can be lacking. Furthermore, NBSs have been argued to neglect to take into account local or indigenous knowledge, often termed Traditional Ecological Knowledge (TEK) in their implementation [22]. These shortcomings can result in unintended consequences, including vulnerabilities in NBS settings [23], as we explore in detail in this paper.

### 3.2. Treescapes

Treescapes refer to all environments predominantly characterised by groups or assemblages of trees, encompassing a diverse range of settings, including traditional woodlands, small urban parks, clusters of trees within cities, tree-lined streets, and roadside tree groupings. This concept extends beyond the boundaries of conventional forests to encompass both natural and human-made green spaces, where trees collectively create significant ecological and social value. In this article, “treescapes” include urban, peri-urban and rural woodlands (including community-owned woodlands), urban green pockets, roadside tree corridors, public parks, and any landscape where trees form a visible or functional network that supports biodiversity, ecosystem services, and community well-being [24,25].

### 3.3. Biogeographical Regions

Biogeographical regions, or bioregions, delineate species pools that differ in their historical origins, taxonomic composition, and ecological and evolutionary attributes [26]. They constitute large-scale divisions of Europe that represent areas with distinct evolutionary histories, each containing unique flora and fauna [27]. This differentiation arises because habitats and species face varied challenges across Europe, influenced by diverse factors such as climate, topography, and soil conditions [28]. Biogeographical regions profoundly influence communities by shaping resource availability, economic activities, cultural practices, and the overall quality of life. Environmental factors collectively shape cultural practices and social structures, underscoring the integral role of biogeographical regions in the development and well-being of societies. Community challenges within biogeographical areas are complex and intricately connected to various environmental, economic, and cultural elements [29]. The biogeographical regions of Europe are diverse and have been extensively studied to understand their ecological and environmental significance. Case study assignments were conducted at the country level based on the official biogeographical regions map from the European Environment Agency. Because some European countries span multiple biogeographical regions, and precise spatial coordinate matching was not available across all repository databases, this country-level classification introduces a degree of spatial uncertainty, which is acknowledged as a methodological limitation. The distribution of European countries across these distinct zones is detailed in Table 1.

## 4. Methods

### 4.1. Overview

**Figure 1:**
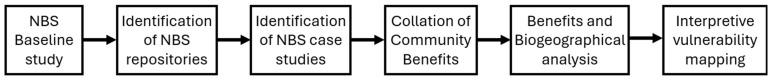
Reverse-logic methodology for exploratory vulnerability identification, illustrating six analytical phases from baseline NBS case analysis through interpretive vulnerability mapping across European treescapes. The horizontal arrows indicate the directional, progressive flow of the qualitative data transformation process.

This study employed a multi-stage, reverse-logic analytical framework to identify and analyse 131 treescape NBS case studies across European biogeographical regions, transforming community benefits into inferred underlying vulnerabilities. The methodological approach was structured around six sequential phases: (1) conceptual framework development through baseline case study analysis, (2) systematic identification of European NBS repositories, (3) treescape case study selection using adapted Preferred Reporting Items for Systematic Reviews and Meta-Analyses (PRISMA) guidelines, (4) collation and thematic categorisation of community benefits, (5) biogeographical distribution analysis, and (6) interpretive vulnerability mapping. The sequential progression of these six analytical phases and the operational workflow are illustrated in Figure 1.

### 4.2. Multi-Phase Design

The research design integrated evidence-based innovative reverse-logic analysis to bridge the gap between documented NBS benefits and theoretical vulnerability frameworks. Following established systematic review protocols [31,32], the methodology was structured to ensure transparency, reproducibility, and scientific rigour while accommodating the novel reverse-engineering approach required to transform benefit data into vulnerability insights.

#### 4.2.1. Phase 1: Conceptual Framework Development

Initial framework development utilised a baseline case study of Murray Park, a 31-hectare mixed community-owned woodland in Alford, Scotland, to establish methodological parameters and selection criteria. Primary qualitative data from 13 interviews and 64 surveys conducted between November and December 2023 provided empirical grounding for four key constructs: urban connectivity, community involvement, ecological diversity, and measurable benefits. This baseline investigation informed subsequent database search strategies, Boolean operator construction, and comparative assessment frameworks necessary for systematically identifying cases across European repositories.

#### 4.2.2. Phase 2: Systematic NBS Repository Identification

A comprehensive search strategy was employed using the Web of Science and Google Scholar databases to map and catalogue 13 primary NBS repositories cited in the academic literature. The systematic review utilised structured search strings incorporating Boolean operators to identify European-funded programmes maintaining treescape case study databases. The snowball sampling methodology, initiated from peer-reviewed articles, enabled the identification of both academic repositories and the grey literature sources containing relevant NBS documentation spanning the period from 2015 to 2024. The specific structured search strings and Boolean parameters utilised for database querying are detailed in Table 2 for the Web of Science and Table 3 for Google Scholar.

To identify relevant case studies, the research drew upon the peer-reviewed literature retrieved from the academic databases Web of Science and Google Scholar. These sources were systematically examined to identify reputable online databases and repositories that contain documented NBS case studies, with a specific focus on those associated with European programmes and initiatives.

In several instances, a snowball sampling approach was employed, beginning with key references identified in the academic literature, to trace additional NBS cases across related databases and project repositories. This iterative method helped ensure the inclusion of diverse, contextually relevant examples that captured the breadth of NBS implementation across Europe. The search for case studies was guided by a purposefully designed search string, tailored to capture variations in terminology related to NBS, ecosystem-based adaptation, and community resilience.

#### 4.2.3. Phase 3: PRISMA-Guided Case Study Selection

Repository screening and case study selection followed an adapted Preferred Reporting Items for PRISMA framework, modified to accommodate the synthesis of established database content rather than traditional literature screening. Searches were conducted between January and June 2024. The comprehensive search specifically targeted databases containing treescape NBS case studies that met four inclusion criteria: urban area linkage, community involvement, ecological diversity, and documentation of measurable benefits. Following systematic screening protocols, 131 case studies were selected from repositories across European biogeographical regions, with exclusion criteria systematically recorded to maintain methodological transparency.

The English-language peer-reviewed and grey literature were reviewed. Publication dates from 2015 to 2024 were considered. The review intentionally excludes treescape areas used for timber production to maintain a focus on community-centric benefits rather than economic or industrial outputs. It also omits specific ecosystem services, such as carbon sequestration and water purification, that do not directly relate to community engagement or well-being. The treescape screening criteria included woodlands, rural and peri-urban forests, nature reserves, riparian zones, large public parks, urban wooded green spaces, restored habitats, and tree-covered catchment areas. NBS woodlands were considered if they primarily emphasise developing nature, integrating ecology into development, promoting ecological systems, and linking to people, place, and landscape, as well as water and terrestrial resource management. Additional community benefits in Table 2 included tourism support, health-driven eco-tourism and noise reduction. Exclusion criteria included water purification and retention, carbon sequestration, coastal resilience and improving property value.

The review was guided by PRISMA framework, with selected elements adapted to suit the synthesis of data from established online databases and repositories. The comprehensive search targets explicitly the identification of databases and repositories containing relevant NBS case studies.

Following collation, all records were screened for relevance. Case studies meeting initial inclusion criteria were retrieved and examined for suitability. Records were excluded if they were duplicates, inaccessible, outside of Europe, or lacked a substantial community-based treescape component; reasons for exclusion were systematically recorded. The final selection of case studies was subjected to in-depth qualitative analysis to ensure methodological transparency and rigour. The final distribution of the selected case studies across their respective European research programmes and online repositories is synthesised in Table 4.

**Figure 2:**
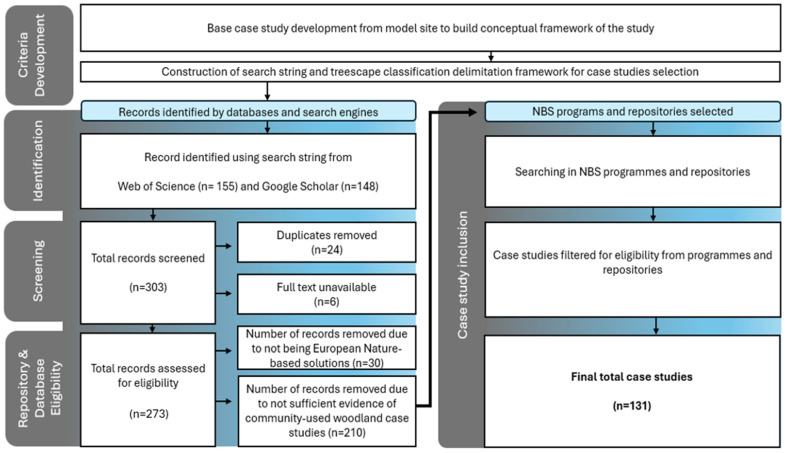
Research process flowchart, including the systematic literature review and the identification of regional repositories and databases for potential case study selection. The vertical layout columns denote consecutive methodological stages running from criteria development down to final sample inclusion, whilst the blue-shaded header blocks highlight the initial database retrieval streams. The directional black arrows trace the exact filtration pathways and sample attrition mechanics.

The selection process for NBS programmes and repositories prioritised both the relevance and comprehensiveness of the treescape descriptions. The resulting set of studies encompassed a broad cross-section of biogeographical regions, environmental contexts, and diverse NBS types, including urban parks, reserves, green corridors, national parks, nature reserves, and food forests, providing a holistic overview of NBS implementation and benefits across Europe. The step-by-step attrition strategy, repository screening, and database filtering protocols that determined this final case study sample size are illustrated in Figure 2.

#### 4.2.4. Phase 4: Thematic Benefit Categorisation

Community benefits identification utilised standardised categorisation systems embedded within European-funded NBS platforms, ensuring consistency and comparability across projects. The 131 selected case studies generated coded benefits distributed across standardised benefit menus that project contributors used when uploading case study details. Thematic qualitative analysis distilled these benefits into main benefit categories.

#### 4.2.5. Phase 5: Biogeographical Distribution Analysis

Spatial analysis mapped the distribution of case studies across European biogeographical regions, examining how the manifestation of benefits varies according to local climatic stressors, ecological characteristics, and socio-economic contexts. The regional analysis utilised a Sankey diagram to illustrate the flow of benefits between biogeographical regions and thematic categories, revealing how environmental pressures and community needs shape NBS benefit prioritisation across Europe’s varied landscapes.

#### 4.2.6. Phase 6: Reverse-Logic Vulnerability Inference

The methodology’s innovative reverse-logic approach systematically transformed identified community benefits into theoretically underlying vulnerabilities that necessitate NBS interventions. This analytical framework operated on the assumption that benefit prominence and distribution patterns reflect corresponding community and ecosystem vulnerabilities that treescape NBSs are designed to help address. Where traditional approaches begin with vulnerability identification and assess the effectiveness of interventions, this reverse-engineering methodology starts with documented benefits. It infers the vulnerability context that renders these benefits essential.

The reverse-logic transformation process involved five systematic steps: (1) quantifying benefit frequency and distribution across biogeographical regions, (2) identifying benefit clusters that suggest underlying vulnerability themes, (3) theoretically extrapolating community and ecosystem vulnerabilities that would necessitate observed benefit patterns, (4) cross-referencing inferred vulnerabilities with regional climatic and socio-economic stressors, and (5) synthesising vulnerability determinants of vulnerability scenarios.

It is important to distinguish between empirically ‘observed benefits’ extracted from the case studies and the theoretically ‘inferred vulnerabilities’. To mitigate the risk of over-inference, the inferred vulnerabilities presented in this framework serve as theoretical indicators. In practice, these inferences should be paired with external validation, such as regional demographic statistics or local health policy documents, to strengthen their credibility. To demonstrate methodological transparency, the mapping rules operated on direct thematic correspondence (e.g., the documented benefit of ‘reducing urban heat island effects’ was mapped directly to the indicative vulnerability of ‘urban heat and local climate destabilisation’, which fed into the broader grouping of ‘Physical/Environment Security’).

#### 4.2.7. Derivation of Vulnerability Scenarios (Clusters)

Vulnerability assessment within NBS treescape research necessitates a comprehensive framework that moves beyond singular, isolated vulnerability factors to embrace the complex interconnectedness of socio-ecological systems. This study employed a structured clustering methodology to systematically group vulnerabilities that exhibit similar thematic contexts and operational characteristics, thereby enabling more targeted and practical analysis of the multidimensional challenges that influence treescape benefits.

The first step was to identify corresponding indicative individual vulnerabilities for each benefit, then organise them into thematic clusters based on their functional relationships and overlapping influences on socio-ecological systems. This clustering approach recognises that vulnerabilities do not operate in isolation but rather form interconnected networks of risk that simultaneously affect multiple dimensions of community well-being and environmental health.

## 5. Results and Discussion

### 5.1. Structural Classification of Case Studies

The first phase of the systematic review revealed that municipal parks are the dominant typology of treescape-related NBSs, reflecting both their accessibility to the public and their feasibility within established urban planning frameworks. This predominance highlights the central role of local governments in driving tree-based interventions in and around urban areas, as well as their partnerships with NBS projects. Nevertheless, the presence of more specialised approaches, such as food forests, hydrological interventions, and restoration projects, indicates a growing diversification of practices tailored to regional and environmental contexts. A comprehensive inventory and structural typology of the 131 examined nature-based solution treescape settings are summarised in Table 5.

### 5.2. What Categories of Community Benefits Are Most Frequently Identified in European Treescape NBS Databases and Repositories?

The community benefits presented in this study were derived from benefit categories identified within the selected NBS databases and repositories. When contributors upload case study details to these European-funded NBS platforms, they are provided with standardised benefit selection menus to ensure consistency and comparability across projects. The benefit categories listed below represent the most frequently selected options by project contributors when describing their treescape NBS initiatives. This standardised categorisation system enables meaningful comparison across the 131 case studies while capturing the diverse ways that treescape NBSs deliver community value across different European biogeographical regions. A detailed synthesis of these main classifications alongside their corresponding repository parameters is compiled in Table 6.

### 5.3. How Are the Community Benefits of Treescape NBS Projects in Europe Distributed Across Biogeographical Regions?

This data from the online repositories highlighted regional differences in the benefits communities derive from treescape NBS across Europe, shaped by biogeographical, cultural, and socio-economic contexts. Recreation emerged as the most widely reported benefit, particularly in urban and peri-urban woodlands, where it supports physical and mental health by providing accessible spaces for leisure and relaxation. In Atlantic and Continental regions, social cohesion and accessibility were emphasised, reflecting efforts to foster inclusivity and community connection. Conversely, Alpine areas prioritised hazard mitigation, such as reducing landslide and avalanche risks.

From a transitional vulnerability perspective, the concentration of benefits in temperate regions creates potential risks during environmental and policy transitions. The heavy reliance on Continental, Mediterranean, and Atlantic treescapes for community services means that climate change impacts on these regions could have a disproportionately negative effect on human well-being. Mediterranean forests are particularly vulnerable to increased drought and fire risk, which could disrupt recreational and cultural services and create socio-economic vulnerabilities.

Cultural ecosystem services, such as recreation, education, and mental health support, were predominant across all regions, showcasing the integral role of treescapes in enhancing well-being and fostering environmental awareness. However, other benefits, such as food provisioning and spiritual connections, were less commonly reported, reflecting varying cultural practices and urbanisation trends. The study underscores the need for tailored, place-based approaches to treescape NBS design and management, ensuring interventions address specific regional needs and maximise community impact. This approach is essential for harnessing the full potential of treescapes in supporting climate resilience, social equity, and human well-being.

Areas including the Alpine, Pannonian, and Black Sea regions account for just 5.4% of documented benefits, suggesting that extreme environmental conditions or limited accessibility reduce the diversity of documented community benefits. The Alpine region’s low contribution (13 benefits) contrasts with its known biodiversity importance, potentially indicating that steep terrain and harsh climate limit community access to forest benefits.

**Figure 3:**
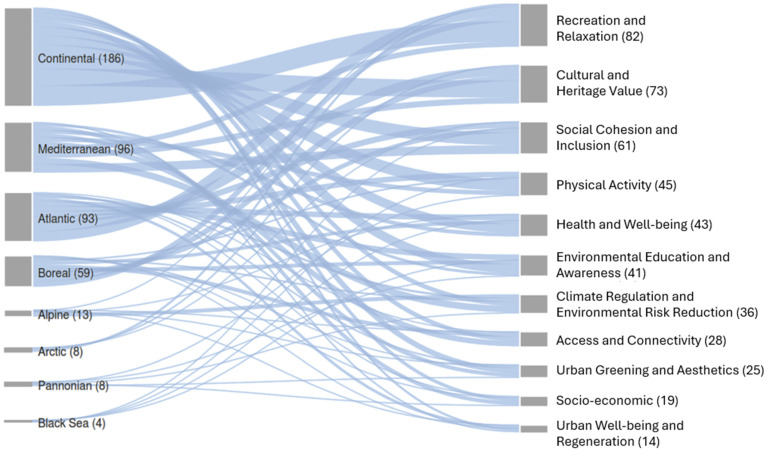
Sankey diagram linking biogeographical regions with community-benefit thematic categories across 131 case studies (*N* = 467 coded benefits). The width of the regional and thematic nodes represents the total accumulated benefits per category, whilst the varying thickness of the connecting paths indicates the exact frequency flow of benefits linking specific bioregions to their corresponding themes.

Data were available for 8 of the 11 European biogeographical regions. Node widths represent total benefits per region or category, while flow widths indicate the number of benefits connecting regions to themes. The largest contributions come from the Continental region (186; 39.8%), followed by the Mediterranean (96; 20.6%), Atlantic (93; 19.9%), and Boreal (59; 12.6%). Smaller shares are represented by the Alpine (13; 2.8%), Arctic (8; 1.7%), Pannonian (8; 1.7%), and Black Sea (4; 0.9%) regions. The most frequently coded themes are Recreation and Relaxation (82; 17.6%), Cultural and Heritage Value (73; 15.6%), and Social Cohesion and Inclusion (61; 13.1%), followed by Physical Activity (45; 9.6%), Health and Well-being (43; 9.2%), and Environmental Education and Awareness (41; 8.8%). The regional distribution of these characteristics is illustrated in Figure 3.

### 5.4. What Are the Underlying Community and Ecosystem Vulnerabilities That the Benefits Associated with Treescape NBS Address?

This section theoretically extrapolates the underlying vulnerabilities that would necessitate the benefits identified in Section 5.3. The analytical framework operates on the premise that the distribution of NBS benefits across European biogeographical regions serves as an indicator of corresponding community and ecosystem vulnerabilities that treescape interventions address.

To reduce subjectivity in forming these five clusters, the derivation followed a systematic thematic analysis process. The coding scheme grouped raw vulnerability indicators into overarching themes based on shared socio-ecological stressors. To enhance objectivity and replicability, the assignment of vulnerabilities to specific clusters was cross-examined by multiple researchers to ensure coder agreement.

The vulnerability clustering methodology categorises specific risks by their primary impact domains: access to natural resources, individual and collective well-being, cultural continuity, ecological literacy, environmental health, and fundamental security needs. Each cluster represents a constellation of related vulnerabilities that share common causative mechanisms and require similar intervention approaches through nature-based solutions.

**Figure 4:**
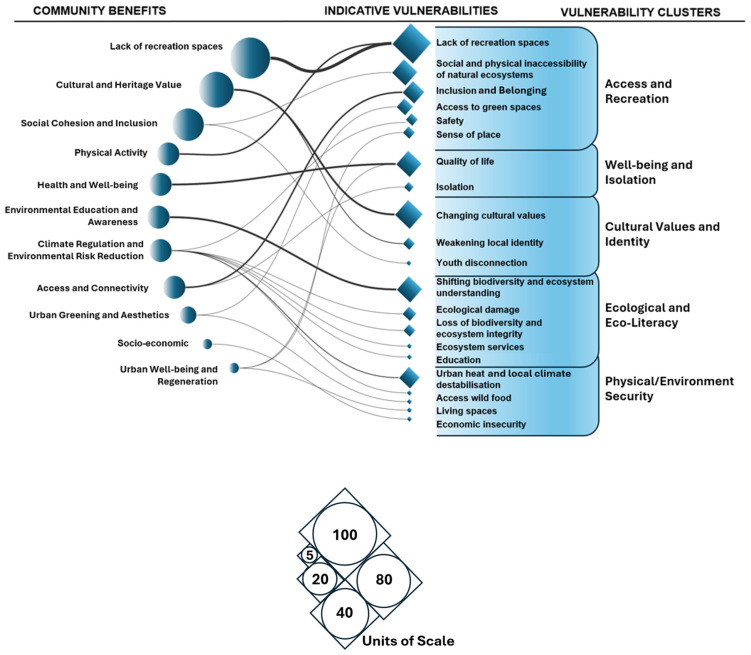
Indicators of NBS treescape community benefits, indicative vulnerabilities, and clustered vulnerability groupings. The structural network diagram illustrates the qualitative mapping paths connecting empirical observations to theoretical risk themes. The varying sizes of the circles and diamonds denote the relative coding frequencies of specific benefits and vulnerabilities within the dataset, whilst the thickness of the connecting lines indicates the strength and frequency of the mapping pathways between them. The concentric bubbles within the ‘Units of Scale’ legend provide a calibrated visual index for these proportional weights.

The five thematic vulnerability groupings discussed below represent the grouping of the indicative vulnerabilities; these do not constitute an exhaustive inventory of all potential vulnerabilities that treescape NBS interventions may address. Rather, they provide a framework for understanding how the benefits of urban treescapes correspond to underlying community and ecosystem needs across European contexts. The conceptual mapping pathways linking specific community benefit observations to indicative vulnerabilities, alongside their relative quantitative weights and overarching analytical clusters, are illustrated in Figure 4.

(1) Access and Recreation—Clustered vulnerabilities related to physical and social barriers to natural spaces, recognising that issues like inaccessibility, lack of recreation spaces, and safety concerns all fundamentally limit people’s ability to engage with and benefit from natural environments.
(2) Well-being and Isolation—This cluster captures vulnerabilities affecting individual psychological and social health, grouping isolation, quality of life concerns, inclusion challenges, and sense of place issues as interconnected factors that influence personal well-being.
(3) Cultural Values and Identity—Identified that vulnerabilities affecting cultural continuity, local identity, and intergenerational connection represent threats to community cohesion and cultural sustainability.
(4) Ecological and Eco-literacy—This cluster addresses knowledge and environmental system vulnerabilities, grouping biodiversity loss, ecosystem damage, and education gaps as interconnected threats to ecological understanding and environmental health.
(5) Physical/Environment Security—Clustered vulnerabilities related to material and environmental stability, recognising that climate destabilisation, soil instability, food security, and economic insecurity all represent threats to basic security and livelihood.

#### 5.4.1. Access and Recreation

The importance of access and recreation could stem from urban communities’ increased need or lack of equitable access to quality recreational spaces, creating disparities for marginalised populations. Inadequate green infrastructure and spatial segregation isolate vulnerable groups from the benefits of nature. These barriers may limit physical activity, mental restoration, and community interaction, all of which are essential for well-being. Urban treescapes, designed as NBSs, alleviate vulnerabilities stemming from limited access to green spaces and recreational opportunities in cities [33,34]. Through urban green infrastructure design that enhances accessibility, including green spaces that meet the expectations and needs of potentially vulnerable groups such as people with disabilities, children, and older adults, physical and psychological barriers to access can be addressed [35,36]. Green spaces with diverse attributes such as landscaped features, ponds, trees, and lakes encourage higher levels of use and facilitate diverse recreational activities including walking, running, cycling, picnicking, and other nature-based activities, while green infrastructure’s networking approach enhances connectivity and accessibility, supporting inclusive participation across different community groups [37,38]. Well-maintained areas with seating, large trees, and designated communication zones for meetings and social gatherings support recreational benefits and social interaction, while addressing disparities in urban green spaces provision that may affect specific population groups differently based on their cultural preferences and demographic characteristics [39].

#### 5.4.2. Well-Being and Isolation

Urban environments may undermine physical and mental health through environmental stressors such as heat, pollution, and limited access to green spaces, potentially increasing social isolation, particularly among vulnerable groups. Rural areas are also experiencing rising levels of loneliness and isolation, especially among older residents, due to the ageing demographic trend present in many such regions [40]. Urban treescapes as an NBS can provide regulating ecosystem services, including urban cooling and air quality regulation, alongside community benefits that support community cohesion and address these vulnerabilities [37,41]. Exposure to nature-based interventions has been shown to benefit cardiovascular health outcomes, including reductions in blood pressure and heart rate. At the same time, forests and woodlands provide well-being benefits encompassing health, connections to nature and landscapes, and stress reduction for individuals across different community groups, though these benefits are unequally distributed across British Society [9,42]. Green spaces such as canopies, parks, and recreation areas help cool cities and reduce exposure to urban heat, which disproportionately affects vulnerable populations [43,44]. Importantly, green spaces in towns and cities foster social inclusion by facilitating social interaction, communal activities, and group activities, thereby reducing isolation and promoting community cohesion [39]. Green space can bring people together from different social groups who may not normally interact, particularly benefiting groups vulnerable to social exclusion, such as people with disabilities, ethnic minorities, young people, older people, and those at an economic disadvantage, while increasing social activity and improving community cohesion in deprived communities [37].

#### 5.4.3. Cultural Values and Identity

The significance of cultural values may emerge from the threat of urbanisation to cultural landscapes and traditional ecological knowledge that anchor community identity. Loss of traditions and intergenerational knowledge transfer may weaken the cultural fabric that supports environmental adaptation. Cultural displacement may reduce connection to place-based identity and historical environmental stewardship practices. Recognition of different needs, demands, values and preferences across social groups in outdoor recreation opportunities supports a sense of belonging and social cohesion amid growing cultural diversity [38]. Urban treescapes become spaces where cultural richness is promoted, traditions are maintained, and new forms of cultural expression are fostered through engagement with youth and diverse populations [36]. This aligns with supporting communities through nature-based interventions that strengthen social infrastructure and foster a sense of place, thereby sustaining virtuous cycles of community engagement over time amid social and environmental change [41]. Such NBSs contribute to recreational opportunities and educational benefits while supporting biodiversity and ecosystem services that help communities build resilience to climate change through restoration and conservation practices [45].

#### 5.4.4. Ecological and Eco-Literacy

Low ecological literacy, particularly among urban populations, could diminish awareness of human-environment interdependencies. This disconnection may create vulnerabilities to environmental change and reduce collective capacity for ecosystem stewardship. Desmarais [46] examines the role of ecological literacy in enhancing ecosystem resilience and reducing environmental degradation, noting that “the general purpose of ecological literacy is to forge comprehension of social and ecological systems, increase awareness of current ecological issues and their interrelations with human societies and provide opportunities for action-oriented learning to take place.” Suwandi et al. [47] argue that schools must foster environmental awareness through teaching materials, asserting that “Environmental education is an important factor to minimise environmental damage”. Part of this cluster is understanding that the core component of urban green management planning is recognising that public urban green areas guarantee an adequate quality of life by providing ecosystem services, including habitat for biodiversity, air purification, runoff mitigation, moderation of environmental extremes, and the transformation of industrial brownfield sites into multifunctional green spaces—to ensure a healthy urban ecosystem [48]. Concurrently, demonstration sites for Urban Green Infrastructure showcase multifunctional benefits, such as food production, recreation, and climate regulation, in real-world settings, while enabling shared learning through hands-on participation, workshops, community events, and volunteer activities that build local skills and foster mutual exchange about caring for nature [35]. As such, treescapes are instrumental not only in direct ecological improvement but also in cultivating stewardship and responsibility among community residents, strengthening socio-ecological ties, and equipping urban populations to participate in future-oriented, sustainable city making.

#### 5.4.5. Physical/Environmental Security

The priority of physical security could be in response to communities’ increasing exposure to extreme weather, flooding, and urban heat resulting from climate change, which threaten safety and liveability. Poor infrastructure maintenance and unsafe pathways might limit outdoor space use for vulnerable populations. Climate change may intensify these risks while exposing the inadequacy of conventional infrastructure protection systems. Urban treescapes mitigate vulnerabilities associated with physical and environmental risks in densely built settings [49]. Key benefits include flood prevention, reduction in landslides and debris flows, control of extreme temperatures, and the creation of shaded, cool refuges during heatwaves [43]. These functions are underscored by reductions in urban heat islands, improvements in water quality, and air pollutant filtering, which directly contribute to safer and more resilient urban environments [41,50]. Improved lighting, secure pathways, and enhanced connectivity of spaces help address fears of crime and actual safety hazards [35]. Moreover, the maintenance of ecosystem services by treescapes provides critical buffers against both everyday and extreme environmental threats, supporting the continued liveability and sustainability of urban, peri-urban, and rural regions [45]. In doing so, NBS transforms vulnerable residential zones into protected, adaptive environments suited for long-term habitation and well-being.

### 5.5. What Are the Determinants of Vulnerability Scenarios (Clusters)?

The study adopts an innovative interpretation of how benefits and vulnerabilities coexist and transform through practice. This section examines the potential drivers of the vulnerabilities identified in Section 5.4. In urban contexts, urban heat islands and water scarcity compromise thermal comfort and public health, threatening broader aspects of environmental well-being. Expanding continuous canopy cover and green corridors mitigates these pressures by reducing surface temperatures through shading and evapotranspiration, while also sustaining recreational and social spaces during hot, dry periods. By alleviating heat stress, such nature-based interventions preserve the use of outdoor amenities and support residents’ mental well-being and social connectivity. Recent findings confirm that human-induced climate change is driving global warming, with the past decade the warmest on record [51]. The primary driver of this vulnerability is the accelerating influence of climate change, which intensifies temperature extremes, lengthens heatwave duration, and increases drought frequency. As global temperatures continue to rise, these climatic shifts redefine baseline residential vulnerability, making adaptive greening strategies increasingly essential in residential areas to safeguard human and ecological resilience in warming environments. Infrastructure resilience and air quality conditions create compound vulnerabilities. Flood risk levels, drought vulnerability, and soil degradation interact to determine how effectively NBS can provide protective ecosystem services.

Communities face geomorphological hazards that threaten habitat integrity and human safety. Protective forest belts serve as living engineering solutions, stabilising slopes and attenuating snow movement to safeguard biodiversity-rich mountain streams and upland meadows. Maintaining these belts ensures the continued delivery of vital ecosystem regulation services while simultaneously enhancing prospects for nature-based tourism, which supports local livelihoods. As visitor numbers rise, particularly in sensitive highland areas, sustainable management and improved protection strategies have become increasingly necessary to mitigate human impacts and safeguard both ecosystem health and visitor safety. The growing popularity of eco-tourism brings new economic opportunities but also heightens the need for robust conservation approaches, responsible infrastructure, and visitor guidance measures to preserve fragile environments and maintain the resilience of habitats, such as alpine social–ecological systems, in the face of intensified human traffic and global change.

Governance and policy frameworks serve as overarching determinants that can either exacerbate or mitigate vulnerabilities across all clusters. Cultural heritage policies and community participation levels serve as critical determinants. When local governance structures fail to include diverse community voices in NBS planning, it can result in solutions that do not reflect local cultural values or traditional ecological knowledge. Vulnerabilities related to the social and physical inaccessibility of natural ecosystems and the lack of recreational spaces stem from socio-economic inequality patterns across Europe. Research demonstrates that socially vulnerable urban neighbourhoods consistently experience lower forest cover and reduced access to quality green spaces. Vulnerabilities related to quality of life, isolation, and sense of place are fundamentally driven by social vulnerability indices that intersect with demographic factors. Age demographics play a crucial role, with elderly populations and children being particularly vulnerable to the adverse effects of limited access to nature, even in rural areas (such as older adults who are unable to access their local woodland due to a lack of accessible pathways). Vulnerabilities related to shifting biodiversity understanding, biodiversity loss, ecosystem integrity, and ecological damage are driven by educational attainment levels and environmental awareness programmes. The effectiveness of scientific knowledge dissemination determines whether communities understand the ecological functions of their local NBS and can participate meaningfully in conservation efforts.

### 5.6. Methodological Innovation

The novel reverse-logic analytical framework developed for this systematic review introduces a methodological innovation that enables vulnerability assessment through benefit pattern analysis when direct vulnerability data are sparse or inconsistent. The approach maintains systematic review rigour through structured protocols while accommodating the theoretical inference necessary to bridge empirical benefit documentation with vulnerability conceptualisation applied at the end of the process. By systematically transforming coded benefits into five vulnerability categories across European biogeographical regions, the methodology demonstrates how established NBS benefit repositories can inform theoretical understanding of how to address socio-ecological vulnerabilities and drive intervention prioritisation. The comprehensive methodology ensures transparency by systematically documenting search strategies, inclusion/exclusion criteria, and analytical frameworks, while the reverse-logic approach provides replicable protocols for applying vulnerability insights across diverse geographical and socio-economic contexts.

### 5.7. Methodological Limitations

The apparent clustering of NBS treescape projects in particular European biogeographical regions likely reflects factors such as uneven research capacity, variable funding allocations, divergent environmental conditions, and differing institutional frameworks, rather than actual differences in tree-based intervention activity. Moreover, reliance on English-language repositories and databases may introduce a systematic language bias, underrepresenting projects published in languages other than English. This bias could exaggerate the prominence of Atlantic and Continental region case studies simply because English dominates online documentation and data access, rather than indicating a higher actual density of NBS implementation in those areas. Furthermore, searching exclusively within English-language databases introduces a systematic language bias that severely underrepresents regions like Eastern Europe, Southern Europe, and the Arctic. As a result, the conclusions cannot be reliably generalised to these regions.

## 6. Conclusions

In revealing how 131 treescape NBS projects across eight European biogeographical regions mobilise five interwoven community benefits: environmental regulation, biodiversity enhancement, health and well-being, social cohesion, and cultural renewal, this study underscores the imperative for place-sensitive design that moves beyond technical interventions to address the fundamental socio-ecological vulnerabilities underpinning community resilience. This research demonstrates the transformative potential of reverse-logic analysis in uncovering the hidden vulnerability landscape that drives NBS implementation across Europe. By systematically mapping benefits from established NBS repositories into five vulnerability categories, the study reveals how environmental stressors alone cannot account for uneven outcomes without addressing entrenched social inequities and governance fragmentation.

The analysis advocates a shift from isolated hazard mitigation to integrated, equity-driven interventions that recognise how biogeographical contexts potentially shape vulnerability patterns. The prioritisation of social cohesion and accessibility in the Continental and Atlantic regions reflects deeper vulnerabilities in community fragmentation and spatial segregation, while the Boreal region’s emphasis on diversified forest management addresses vulnerabilities in both ecological resilience and cultural continuity. These spatial patterns demonstrate that effective NBS design must simultaneously address the climatic, ecological, and socio-economic vulnerabilities that create differentiated exposure across European landscapes.

Moreover, by restoring ecological integrity, rehabilitating soils, reconnecting habitats, and improving air quality, NBS become catalysts for addressing the systemic vulnerabilities that conventional infrastructure cannot resolve. This research presents a novel methodological approach to examining the vulnerabilities that NBS projects address through reverse-logic inference, transforming empirical benefit documentation into theoretical insights on vulnerability. This innovation enables vulnerability assessment in contexts where direct vulnerability data remains sparse or inconsistent, providing replicable protocols for understanding how established NBS interventions inform theoretical frameworks for socio-ecological vulnerability across diverse geographical and socio-economic contexts.

A primary limitation of this research is that it currently provides a theoretical framework that lacks deep local context, serving as an exercise in theoretical synthesis. Furthermore, the inferred nature of the vulnerabilities means there may be instances of inaccuracy in which community benefits stem from proactive policy rather than a direct vulnerability response. However, this reverse-logic approach lays critical foundations and replicable protocols for future, highly contextualised research.

Such a holistic approach promises not only to address the symptoms of environmental vulnerability but to contribute to a better understanding of “transformative treescape governance.” This research conceptualises treescapes as socio-ecological infrastructures and indicators that simultaneously regulate environmental processes, support biodiversity conservation, enhance human well-being, foster social cohesion, and preserve cultural identity. By addressing these interconnected dimensions through place-sensitive design and participatory governance, we can strengthen treescape NBSs to foster stronger, more sustainable relationships between people and nature across Europe’s varied biogeographical landscapes.

While the benefits discussed encompass broad socio-ecological improvements, they possess links to core public health issues. For public health policymakers and community health workers, these findings offer actionable insights for disease prevention and addressing health inequalities. Interventions must move beyond general greening and strategically integrate ecological design with community health policies. By adopting place-sensitive, equity-driven treescape designs that respond to regional climatic and health vulnerabilities, practitioners can position NBS as a policy intervention that mitigates public health stressors and strengthens population-level resilience.

## Figures and Tables

**Table 1:** Biogeographical regions and associated countries.

Region	Countries
Alpine	Austria, Bulgaria, Germany, Spain, Finland, France, Italy, Poland, Romania, Sweden, Slovenia, Slovakia, Ukraine, Russia, Georgia, Armenia
Arctic	Iceland, Norway, Russia
Anatolian	Turkey
Atlantic	Belgium, Germany, Denmark, Spain, France, Ireland, Portugal, Netherlands, United Kingdom
Black Sea	Bulgaria, Romania, Turkey, Georgia
Boreal	Estonia, Finland, Latvia, Lithuania, Sweden, Belarus, Russia
Continental	Austria, Belgium, Bulgaria, Czech Republic, Germany, Denmark, France, Italy, Luxembourg, Poland, Romania, Sweden, Slovenia, Belarus, Ukraine, Russia, Moldova, Serbia
Macaronesian	Spain, Portugal
Mediterranean	Cyprus, Spain, France, Greece, Italy, Malta, Portugal, Turkey
Pannonian	Czech Republic, Hungary, Romania, Serbia, Slovakia, Ukraine
Steppic	Romania, Moldova, Ukraine, Russia

Note: Country assignments are based on the official biogeographical regions map from the European Environment Agency, used to allocate countries for NBS case studies. Adapted from European Environment Agency [30] and Cervellini et al. [28].

**Table 2:** Search string used in the Web of Science.

Search String Topic Field		Search String Location Field		Search String Community Benefits
“nature-based solutions” (Abstract)	AND	Europe* OR EU OR Austria OR Belgium OR Bulgaria OR Croatian OR Cyprus OR Czechic OR Denmark OR Estonian OR Finland OR France OR Germany OR Greece OR Hungary OR Iceland OR Italy OR Latvian OR Lithuanian OR Luxembourg OR Malta OR Netherlands OR Poland OR Portugal OR Romanian OR Slovak* OR Slovenian OR Spain OR Sweden OR “United Kingdom” OR UK (Abstract)	AND	access* OR benefit* OR cognitive OR community OR connect* OR emotion* OR exercise* OR heal* OR immune OR job* OR peace OR “physically active*” positivity OR quality OR relax OR religio* OR safety OR space OR societal OR meditat* OR mental OR mindfulness OR “move freely” OR outdoor OR peac* OR prescription OR social OR stress OR spiritual OR therap* OR walk OR well-being OR well-being (All Fields)

* indicates that the wild-card operator is used (e.g., Europe* will identify all terms beginning with the sequence, including Europe and European).

**Table 3:** Search string used in Google Scholar.

Search String All Words		Search String Exact Phrase		Search String in Full Article
forest*	AND	“nature-based solutions”	AND	access* benefits cognitive community connect* exercise* heal* job* peace “physically active” quality relax religio* safety meditat* mindfulness “move freely” prescription social stress spiritual therapy walk well-being well-being

* indicates that the wild-card operator is used (e.g., forest* will identify all terms beginning with sequence, including forested and forests).

**Table 4:** NBS programmes and repositories identified in the systematic review as sources for treescape case studies.

Programmes and Repositories Related to NBS	Number of NBS Treescape Case Studies
AIFORIA	5
CLEARING HOUSE	2
CONNECTING NATURE	6
EUPOLIS	2
GROW GREEN	5
NATURVATION	71
NETWORK NATURE	16
OPPLA	5
PHUSICOS	4
RECONECT	4
UNALAB	3
URBAN GREENUP	3
VARCITIES	2
Additional Snowball Sampling/Grey Literature	3
Total	131 Case Studies

**Table 5:** Typology of NBS treescapes in the case studies.

Afforestation projects: 1	Memorial parks: 1
Alluvial meadows: 1	Municipal parks: 70
Beach parks: 1	National parks: 1
Botanical gardens: 2	Natural life parks: 1
Coastal parks: 1	Natural parks: 3
Conservation areas: 2	Nature reserves: 2
Continuous cover forests: 1	Port ecological areas: 1
Cycle and pedestrian routes: 2	Restoration projects: 3
Eco-districts: 3	River basin management: 2
Educational forest projects: 1	River parks: 1
Environmental parks: 2	Stream restoration projects: 1
Food forests: 2	Tiny forests: 1
Green belts: 2	Tree cadastre systems: 1
Green corridors: 2	Tree strategy systems: 1
Island parks: 2	Urban forests: 5
Landfill restoration: 1	Urban redevelopment projects: 1
Landscape parks: 5	Valley parks: 3
Meadow orchards: 1	Wetlands: 1
Total: 131 case studies	

**Table 6:** Example sub-benefits for each community benefit category.

Community Benefit Category	Example Sub-Benefits
Recreation and Relaxation	Opportunities for relaxation and recreation; Recreation/leisure areas; Festivals, concerts, events; Water recreation
Cultural and Heritage Value	Preservation of natural heritage; Protection of historic/cultural landscapes/infrastructure; Preservation of historic traditions; Promotion of cultural diversity; Contribute to culture and increased cultural richness
Social Cohesion and Inclusion	Social interaction and contact; Social cohesion and inclusion; Social justice and equity; Community engagement and sense of ownership; Social revitalisation
Health and Well-being	Increase well-being and quality of life; improve health (physical and mental); reduce depletion of cognitive resources; promote active, healthy lifestyles
Physical Activity	Cycling/walking/physical activity in green spaces; Playgrounds/trails/agility circuits for children and dogs; Recreation for all age groups (children, elderly, youth-oriented spaces)
Environmental Education and Awareness	Environmental education: Increase awareness and knowledge of NBS; Social learning about NBS, culture, and the importance of green spaces
Climate Regulation and Environmental Risk Reduction	Reducing geo-hazards (rockfall, debris flows, landslides); Reduce heat island effect/cooling spaces/improve ventilation; Provide shade/urban canopies/albedo control; Filtration of ground/air pollutants/enhance the quality of water; Reduce/manage extreme temperatures; Flood prevention/water management; Biodiversity increase/protection
Access and Connectivity	Access to nature; Secure pathways; Shaded pedestrian walkways
Urban Greening and Aesthetics	Green space creation/management; Providing semi-natural green spaces; Increasing green open spaces for residents; Green/eco streets/vertical gardens
Urban Well-being and Regeneration	Restore a brownfield area; Creation of attractive public/liveable/eco-social spaces; Urban densification/improving local living conditions
Socio-economic	Tourism support; Visitor opportunities; Tourism activities; Job creation and employment

## Data Availability

The original contributions presented in this study are included in the article. Further inquiries can be directed to the corresponding authors.
